# Potential drug–drug interactions of frequently prescribed medications in long COVID detected by two electronic databases

**DOI:** 10.1371/journal.pone.0293866

**Published:** 2023-11-16

**Authors:** Theejutha Meakleartmongkol, Supawit Tangpanithandee, Natcha Vanavivit, Apisada Jiso, Pisut Pongchaikul, Suppachok Kirdlarp, Phisit Khemawoot, Surakit Nathisuwan

**Affiliations:** 1 Chakri Naruebodindra Medical Institute, Faculty of Medicine Ramathibodi Hospital, Mahidol University, Samut Prakan, Thailand; 2 Department of Pharmacy, Faculty of Pharmacy, Mahidol University, Bangkok, Thailand; University of the Witwatersrand, SOUTH AFRICA

## Abstract

Infection by severe acute respiratory syndrome coronavirus 2 (SARS-CoV-2) leads to a wide range of acute and chronic complications including long COVID, a well-known chronic sequela. Long COVID often necessitates long-term treatment, which may lead to an increased potential for drug–drug interactions (DDIs). The objective of this study was to assess potential DDIs among frequently prescribed medications in long COVID by using two electronic databases. Sixty frequently prescribed agents were selected from Thailand’s National List of Essential Medicine 2022 for potential DDI analysis by Micromedex and Drugs.com. From these databases, 488 potential DDIs were identified. There were 271 and 434 DDI pairs based on Micromedex and Drugs.com, respectively. Among these DDIs, 77 pairs were labeled as contraindicated or major by both databases. The most common mechanisms for these serious interactions are cytochrome P450 (CYP) inhibition (45%), CYP induction (19%), and QT interval prolongation (7.8%). Based on Fleiss’ kappa (0.073), there was only slight agreement of the DDI severity classifications between these two databases. In conclusion, a large number of potential DDIs were detected among frequently prescribed medications for long COVID. Health care providers should be aware of these DDIs, particularly those that are deemed as contraindicated or major. These DDIs are most likely to cause significant adverse events in patients with long COVID because polypharmacy is common.

## Introduction

The coronavirus disease 2019 (COVID-19) is an infectious disease caused by the severe acute respiratory syndrome coronavirus 2 (SARS-CoV-2) [1]. The disease is responsible for the ongoing COVID-19 pandemic that has affected over 769 million and claimed 6.9 million lives as of 7 August 2023 [2]. The symptoms of infection usually occur within the first 4 weeks, but some might persist long after [3]. The post-infection sequelae are sometimes informally referred to as post-COVID complications, one of which is termed ‘long COVID’. In October 2021, the World Health Organization clinically defined long COVID as a condition that occurs in individuals with a history of probable or confirmed SARS-CoV-2 infection (usually within 3 months from the onset of COVID-19). Patients generally have symptoms lasting at least 2 months that cannot be explained by any alternative diagnosis. Common symptoms of long COVID include–but are not limited to–fatigue, shortness of breath, chest pain and tightness, headaches, problems with memory and focus, coughing, diarrhea, changes to senses, and loss of appetite [4]. A recent meta-analysis of over 1.7 million cases of COVID-19 suggested that 43% of infected individuals experienced some form of sequelae [5]. Long COVID has been reported in 54% of hospitalized patients and 34% of non-hospitalized patients [5]. This suggests that the more severe the acute phase is, the more likely it is for patients to develop long COVID. This conclusion is supported by the United States Centers for Disease Control and Prevention [6]. Because the presence of multiple chronic diseases is one of the key risk factors for severe acute infections, this subgroup of patients has a high likelihood of developing long COVID. Long COVID often necessitates long-term treatment, which may lead to an increased potential for drug–drug interactions (DDIs) due to polypharmacy. Therefore, understanding potential DDIs among patients with long COVID is useful to avoid or prevent any adverse events from serious DDIs.

While there are various tools to identify DDIs to warn clinicians, drug interaction databases like Micromedex and Drugs.com are commonly used in daily practice. However, there are discrepancies between the two databases, thus requiring comparison and review to determine the safety of drug co-administrations. As both underlying chronic diseases and long COVID require pharmacological interventions, it is imperative that the list of normally prescribed therapeutic agents for both conditions be cross-checked for potential DDIs. Adequate information would allow physicians to make better-informed decisions regarding the selection and use of pharmacological interventions in patients with long COVID. This study aims to determine potential DDIs among frequently prescribed medications in long COVID as detected by Micromedex and Drugs.com. Agreement of severity classifications and probable mechanisms of potential DDIs reported by the two databases are also summarized to provide appropriate information to both healthcare providers and patients with long COVID.

## Materials and methods

### Drug selection

**Fig 1:**
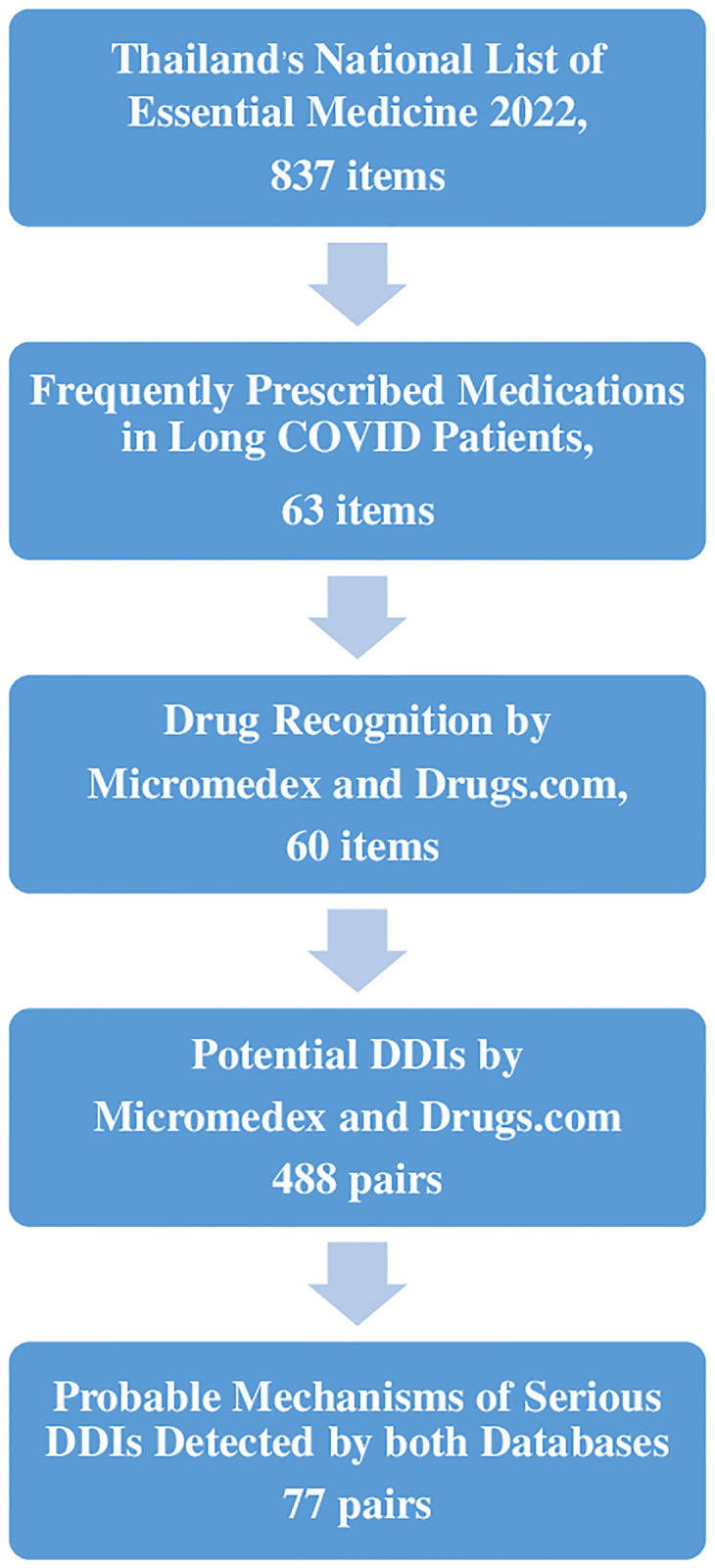
Drug selection, determination of potential drug–drug interactions (DDIs), and analysis for probable mechanisms of serious DDIs determined by Micromedex and Drugs.com.

The focus of this study was on frequently prescribed medications for patients with both long COVID and other common chronic conditions such as diabetes mellitus, hypertension, dyslipidemia, and chronic infections. Medications available to treat these diseases were subsequently chosen from Thailand’s National List of Essential Medicine 2022 and the Chakri Naruebodindra Medical Institute (CNMI) drug lists–accessed in July 2023. A total of 63 agents were chosen by an infectious specialist and immunology team, who has major responsibility in conducting clinical care to COVID patients. Three agents, namely manidipine, fenoterol and bromhexine, were not recognized by Drugs.com or Micromedex. Hence, a total of 60 frequently prescribed medications for long COVID were included in the analysis of potential DDIs using Micromedex and Drugs.com in July 2023 (Fig 1 and Table 1). Ethical approval and consent were not required, and no patient assessment or confidential information was collected in this study.

**Table 1:** List of frequently prescribed medications in long COVID.

No.	Group	Drug
1.	Antihypertensive drugs	Amlodipine
2.		Losartan
3.		Enalapril
4.	Lipid-lowering drugs	Atorvastatin
5.		Rosuvastatin
6.		Simvastatin
7.		Pitavastatin
8.	Antiplatelet drugs	Aspirin
9.		Clopidogrel
10.	Antidiabetic drugs	Metformin
11.		Glipizide
12.		Pioglitazone
13.		Sitagliptin
14.		Insulin
15.	Drugs for Benign Prostatic Hyperplasia	Doxazosin
16.		Tamsulosin
17.	Drugs for Chronic Kidney Disease	Calcium Carbonate
18.		Hydralazine
19.	Antituberculous drugs	Isoniazid
20.		Rifampicin
21.		Pyrazinamide
22.		Ethambutol
23.	Antibacterial drugs	Amoxicillin / Clavulanic acid
24.		Levofloxacin
25.		Ciprofloxacin
26.		Sulfamethoxazole / Trimethoprim
27.	Antifungal drugs	Fluconazole
28.		Itraconazole
29.		Voriconazole
30.		Posaconazole
31.	Antivirals	Acyclovir
32.		Ganciclovir
33.		Valganciclovir
34.		Remdesivir
35.		Molnupiravir
36.		Nirmatrelvir/Ritonavir
37.	Anti-HIVs	Tenofovir disoproxil fumarate
38.		Tenofovir alafenamide
39.		Emtricitabine
40.		Zidovudine
41.		Abacavir
42.		Lamivudine
43.		Nevirapine
44.		Efavirenz
45.		Rilpivirine
46.		Dolutegravir
47.		Raltegravir
48.		Darunavir
49.		Ritonavir
50.		Atazanavir
51.	Respiratory drugs	Dextromethorphan
52.		Albuterol
53.		Salmeterol
54.		Fluticasone
55.		Ipratropium Bromide
56.		Budesonide
57.		Acetylcysteine
58.		Cetirizine
59.	Miscellaneous	Lansoprazole
60.		Omeprazole

### Databases

The two databases used in this study are IBM Watson’s Micromedex and Drugs.com. Micromedex is a subscription-based literature database containing in-depth information relating to drugs, diseases, and other clinical tools [7]. Micromedex is a part of the IBM Watson Health company and widely used among various health professionals. Access to the Micromedex database was via a license to Mahidol University (2023). Drugs.com is a free online pharmaceutical database that indexes information from multiple leading databases, including Cerner Multum, the American Society of Health System Pharmacists, and IBM Watson’s Micromedex [8]. Drugs.com is a popular database for patients and the public domain. The site is certified both by TRUSTe online privacy certification and Health on the Net Foundation’s HONcode [8, 9].

### Documentation

Both databases report information regarding potential DDIs with details on severity, the underlying mechanism, clinical management, and references. Severity classification of DDIs varies between the two databases. Micromedex classifies severity into four categories including contraindicated, major, moderate, and minor. Contraindicated typically refers to a situation where the two interacting drugs should not be used concomitantly due to the high likelihood of harm to a patient. Major indicates that the interaction may be life-threatening and/or require medical interventions to minimize or prevent serious adverse effects. Moderate indicates that the interaction may exacerbate the patient’s conditions and/or require a change in therapy. Minor indicates that the interaction has limited clinical effects and no major alteration in drug therapy is required. Drugs.com classifies DDIs severity into three categories: major, moderate, and minor. Major indicates that the interaction will result in significant clinical impacts to the level where the risks outweigh the benefits. Concomitant uses of the two interacting drugs should be avoided. Moderate indicates that the interaction will have moderate clinical significance. Concomitant uses of the combinations can be used only under special circumstances. Minor indicates that the interaction is of minimal clinical significance.

Documentation level was reported by Micromedex as excellent, good, fair, and unknown [7]. Excellent was determined as controlled studies have clearly established the existence of the interaction. Good is defined as that documentation strongly suggests an interaction exists, but well-controlled studies are lacking. Fair is determined as available documentation is poor, but pharmacologic considerations lead clinicians to suspect the interaction exists, or that documentation is good for a pharmacologically similar drug.

### Data analysis

Data were analyzed with Stata 17.0 (StataCorp LLC, USA). Categorical variables are reported as number and percentage. The agreement between databases was determined by Fleiss’ kappa, which can be between -1 and 1. A value of 1 indicates perfect agreement, a value of -1 indicates perfect disagreement, and 0 indicates expected agreement by chance [10]. Kappa values were interpreted via qualitative descriptors for each intra-class correlation values as follows: kappa > 0.80, almost perfect; 0.61–0.80, substantial; 0.41–0.60, moderate; 0.21–0.40; fair, 0.00–0.20, slight; and < 0.00, poor.

## Results

**Fig 2:**
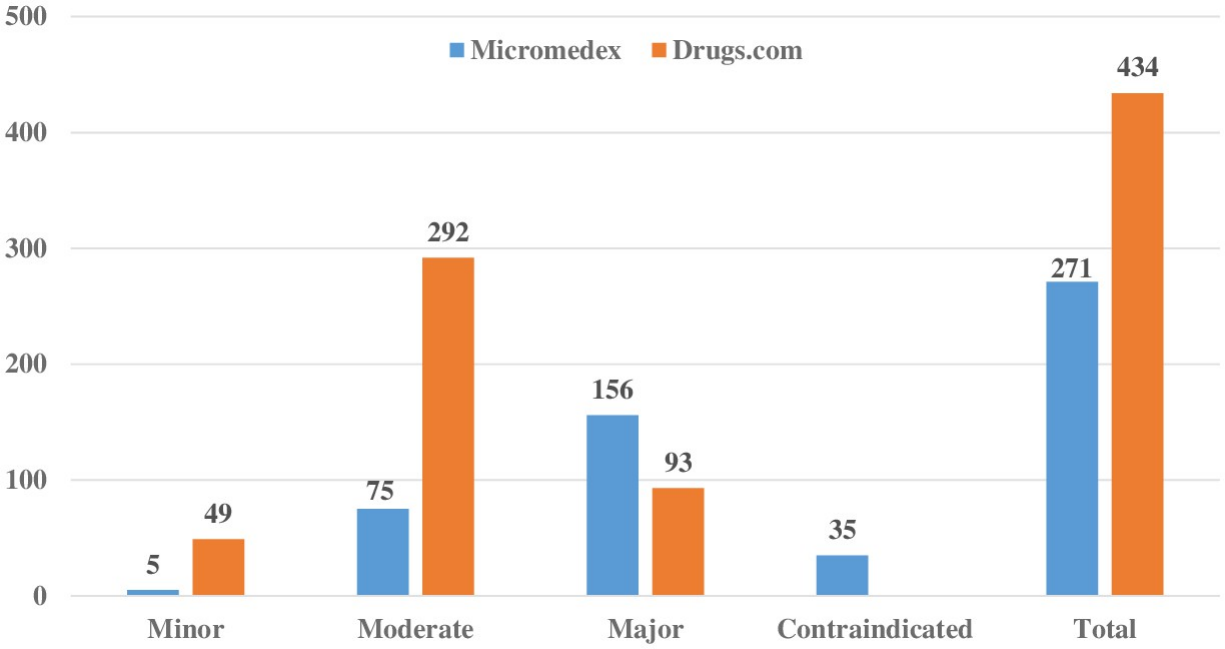
Severity classification of potential drug–drug interactions (DDIs) among frequently prescribed medications in long COVID determined by Micromedex and Drugs.com.

Among the 60 agents of frequently prescribed medications in long COVID, 488 potential DDIs were detected. Micromedex reported 271 DDI pairs, while Drugs.com reported 434 DDI pairs. Of the 271 pairs reported by Micromedex, 35 (12.9%), 156 (57.6%), 75 (27.7%), and 5 (1.8%) were classified as contraindicated, major, moderate, and minor, respectively. Of the 434 pairs reported by Drugs.com, 93 (21.4%), 292 (67.3%), and 49 (11.3%) pairs were considered major, moderate, and minor, respectively. Fig 2 provides details on the severity classification.

**Fig 3:**
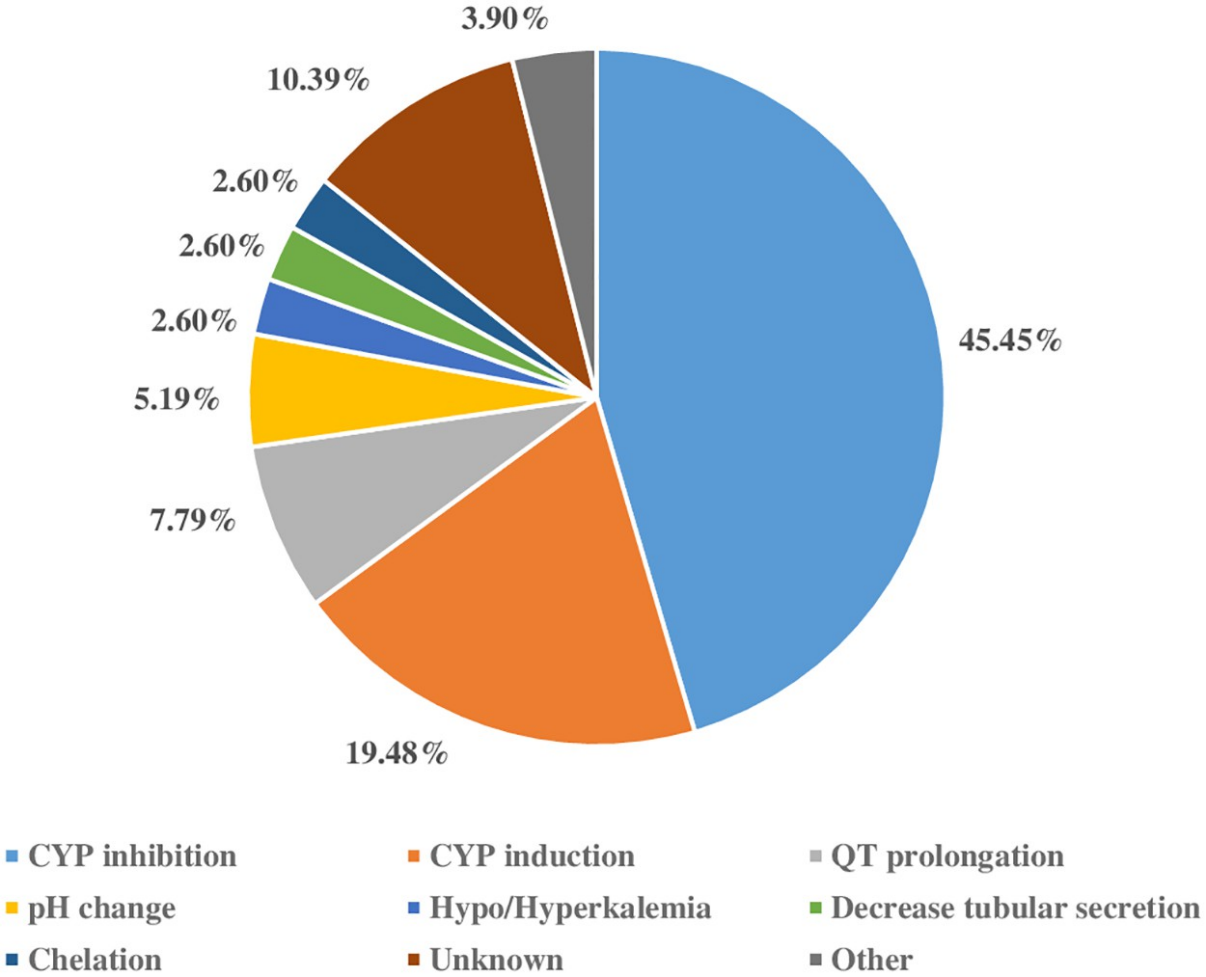
Probable mechanistic of serious drug–drug interactions (DDIs) among frequently prescribed medications in long COVID determined by Micromedex and Drugs.com.

**Fig 4:**
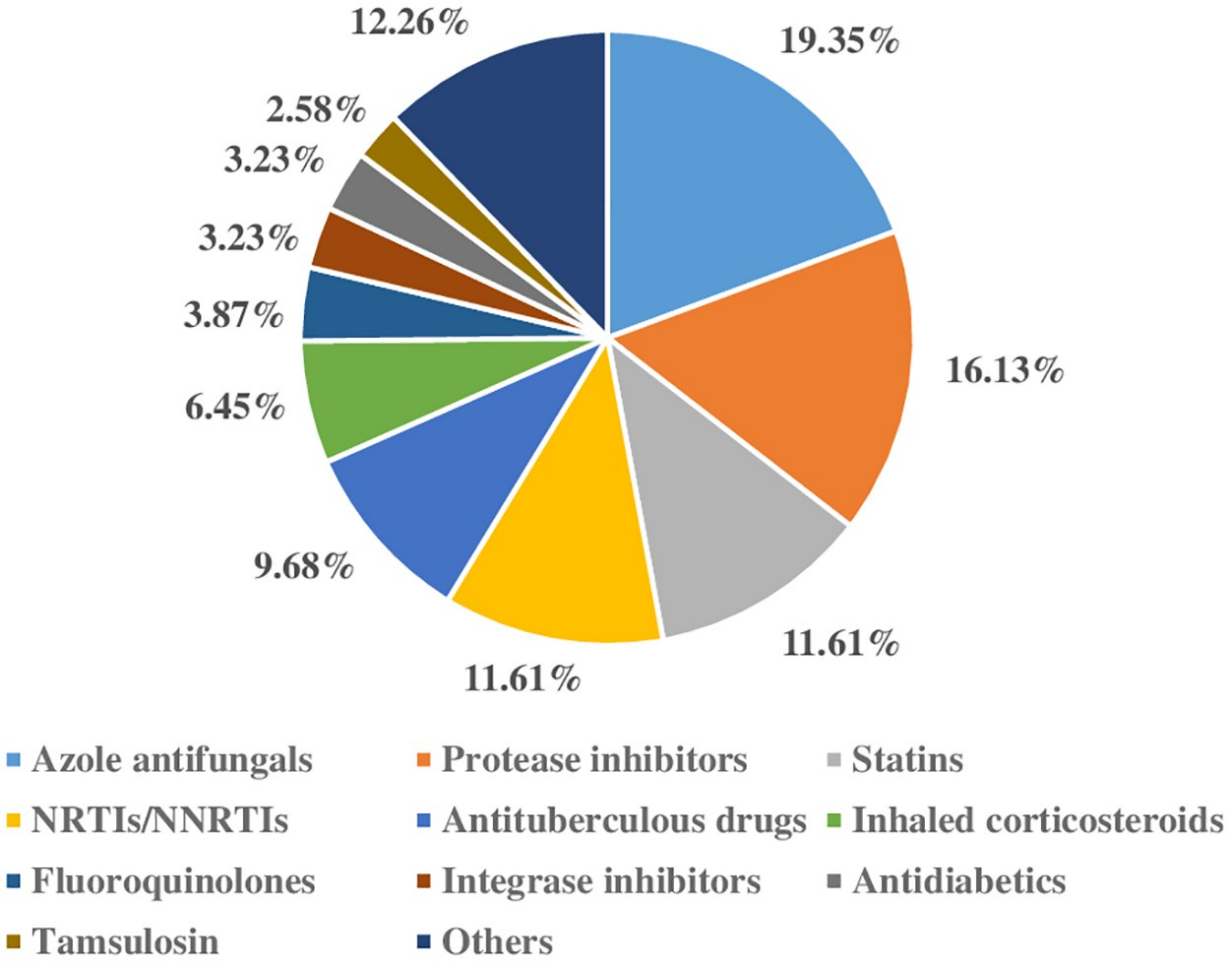
Major classes of medicines cause serious drug–drug interactions (DDIs) among frequently prescribed medications in long COVID determined by Micromedex and Drugs.com.

Of all potential DDI pairs detected, there were 77 severe interactions at the levels of contraindicated or major with high concurrence between the two databases (Table 2). Among serious DDIs reported by both databases, the major mechanistic categories of potential DDIs were cytochrome P450 (CYP) inhibition (45%), CYP induction (19%), and QT interval prolongation (7.8%). Some minor mechanistic categories were gastric pH change, hypo/hyperkalemia, decreased tubular secretion, chelation, and others (Fig 3). Five groups of drugs were commonly implicated: antiretrovirals, antifungals, 3-hydroxy-3-methylglutaryl coenzyme A (HMG CoA) reductase inhibitors or statins, anti-tuberculosis drugs, and corticosteroids (Fig 4). These agents are mainly metabolized by CYPs and/or possess CYP induction and/or inhibitory activities. Documentation levels of potential DDIs as reported by Micromedex were excellent 52 (19.2%), good 66 (24.4%), and fair 153 (56.44%). Potential DDIs with contraindicated or major severity showed higher tendency of excellent or good documentation as reported by Micromedex. Overall, Fleiss’ kappa was 0.073, indicating slight agreement in DDI severity classification between the two databases.

**Table 2:** Severe drug–drug interactions (DDIs) reported with high concurrence between Micromedex and Drugs.com.

No.	Reported DDIs	Probable Mechanisms
1.	Acyclovir-Tenofovir Alafenamide	ACYCLOVIR may result in increased tenofovir alafenamide exposure and an increased risk of tenofovir alafenamide-related adverse effects.
2.	Amlodipine-Itraconazole	ITRACONAZOLE may result in increased amlodipine exposure and an increased risk of congestive heart failure.
3.	Amlodipine-Rifampin	RIFAMPIN may result in reduced amlodipine efficacy.
4.	Amlodipine-Simvastatin	AMLODIPINE may result in increased simvastatin exposure and an increased risk of myopathy or rhabdomyolysis.
5.	Atazanavir Sulfate-Atorvastatin Calcium	ATAZANAVIR may result in increased risk of atorvastatin toxicity, including an increased risk of myopathy or rhabdomyolysis.
6.	Atazanavir Sulfate-Budesonide	ATAZANAVIR may result in increased budesonide exposure.
7.	Atazanavir Sulfate-Efavirenz	EFAVIRENZ may result in decreased atazanavir plasma concentrations and increased risk of QT interval prolongation.
8.	Atazanavir Sulfate-Fluconazole	FLUCONAZOLE may result in increased atazanavir exposure and risk for QT interval prolongation.
9.	Atazanavir Sulfate-Lansoprazole	SELECTED PROTON PUMP INHIBITORS may result in reduced atazanavir exposure and an increased risk of reduced efficacy and development of resistance.
10.	Atazanavir Sulfate-Nevirapine	Concurrent use of ATAZANAVIR and NEVIRAPINE may result in increased risk of virologic failure due to decreased atazanavir exposure; increased risk of nevirapine toxicity.
11.	Atazanavir Sulfate-Omeprazole	SELECTED PROTON PUMP INHIBITORS may result in reduced atazanavir exposure and an increased risk of reduced efficacy and development of resistance.
12.	Atazanavir Sulfate-Rifampin	RIFAMPIN may result in decreased atazanavir plasma concentrations.
13.	Atazanavir Sulfate-Rosuvastatin Calcium	ATAZANAVIR may result in increased rosuvastatin exposure and an increased risk of myopathy or rhabdomyolysis.
14.	Atazanavir Sulfate-Simvastatin	ATAZANAVIR may result in increased risk of simvastatin toxicity, including an increased risk of myopathy or rhabdomyolysis.
15.	Atorvastatin Calcium-Darunavir	DARUNAVIR may result in increased atorvastatin plasma concentrations and an increased risk of myopathy or rhabdomyolysis.
16.	Atorvastatin Calcium-Fluconazole	FLUCONAZOLE may result in increased atorvastatin exposure.
17.	Atorvastatin Calcium-Itraconazole	ITRACONAZOLE may result in increased atorvastatin exposure and an increased risk of myopathy or rhabdomyolysis.
18.	Atorvastatin Calcium-Posaconazole	POSACONAZOLE may result in increased atorvastatin exposure and increased risk of myopathy and rhabdomyolysis.
19.	Atorvastatin Calcium-Ritonavir	RITONAVIR may result in increased atorvastatin exposure and an increased risk of myopathy, including rhabdomyolysis.
20.	Atorvastatin Calcium-Voriconazole	VORICONAZOLE may result in increased atorvastatin exposure and an increased risk of myopathy.
21.	Budesonide-Itraconazole	STRONG CYP3A4 INHIBITORS may result in increased budesonide exposure.
22.	Budesonide-Posaconazole	STRONG CYP3A4 INHIBITORS may result in increased budesonide exposure.
23.	Budesonide-Ritonavir	STRONG CYP3A4 INHIBITORS may result in increased budesonide exposure.
24.	Budesonide-Voriconazole	STRONG CYP3A4 INHIBITORS may result in increased budesonide exposure.
25.	Calcium Carbonate-Dolutegravir Sodium	MEDICATIONS CONTAINING POLYVALENT CATIONS may result in decreased dolutegravir exposure and loss of efficacy.
26.	Calcium Carbonate-Raltegravir Potassium	MEDICATIONS CONTAINING POLYVALENT CATIONS may result in decreased raltegravir concentration.
27.	Ciprofloxacin-Efavirenz	Concurrent use of EFAVIRENZ and QT-PROLONGING DRUGS may result in increased risk of QT interval prolongation.
28.	Ciprofloxacin-Glipizide	Concurrent use of ANTIDIABETIC AGENTS and FLUOROQUINOLONES may result in changes in blood glucose and increased risk of hypoglycemia or hyperglycemia.
29.	Ciprofloxacin-Insulin Pork Regular	Concurrent use of ANTIDIABETIC AGENTS and FLUOROQUINOLONES may result in changes in blood glucose and increased risk of hypoglycemia or hyperglycemia.
30.	Clopidogrel Hydrogen Sulfate-Omeprazole	OMEPRAZOLE may result in reduced clopidogrel’s active metabolite exposure and reduced antiplatelet activity.
31.	Darunavir-Rifampin	RIFAMPIN may result in reduced darunavir exposure and reduced efficacy of darunavir.
32.	Darunavir-Rosuvastatin Calcium	Concurrent use of DARUNAVIR and HMG-COA REDUCTASE INHIBITORS may result in increased statin exposure.
33.	Darunavir-Simvastatin	DARUNAVIR may result in increased simvastatin exposure; increased risk of myopathy or rhabdomyolysis.
34.	Dolutegravir Sodium-Efavirenz	EFAVIRENZ may result in decreased dolutegravir exposure and loss of efficacy.
35.	Dolutegravir Sodium-Nevirapine	NEVIRAPINE may result in decreased dolutegravir exposure with reduced efficacy.
36.	Dolutegravir Sodium-Rifampin	RIFAMPIN may result in decreased dolutegravir exposure and loss of efficacy.
37.	Efavirenz-Fluconazole	Concurrent use of EFAVIRENZ and QT-PROLONGING DRUGS may result in increased risk of QT interval prolongation.
38.	Efavirenz-Levofloxacin	Concurrent use of EFAVIRENZ and QT-PROLONGING DRUGS may result in increased risk of QT interval prolongation.
39.	Efavirenz-Rilpivirine Hydrochloride	EFAVIRENZ may result in reduced rilpivirine plasma concentrations and risk of diminished therapeutic effect of rilpivirine.
40.	Efavirenz-Voriconazole	Concurrent use of EFAVIRENZ and VORICONAZOLE may result in decreased voriconazole plasma concentrations and increased efavirenz plasma concentrations; increased risk of QT prolongation.
41.	Enalapril Maleate-Losartan Potassium	Concurrent use of ANGIOTENSIN CONVERTING ENZYME INHIBITORS and ANGIOTENSIN II RECEPTOR BLOCKERS may result in increased risk of adverse events (ie, hypotension, syncope, hyperkalemia, changes in renal function, acute renal failure).
42.	Enalapril Maleate-Sulfamethoxazole/Trimethoprim	Concurrent use of TRIMETHOPRIM and SELECTED POTASSIUM-SPARING DRUGS may result in increased risk of hyperkalemia.
43.	Fluconazole-Glipizide	Concurrent use of FLUCONAZOLE and GLIPIZIDE may result in increased risk of hypoglycemia.
44.	Fluconazole-Itraconazole	Concurrent use of FLUCONAZOLE and ITRACONAZOLE may result in increased risk of QT interval prolongation.
45.	Fluconazole-Ritonavir	FLUCONAZOLE may result in increased ritonavir exposure and risk for QT interval prolongation.
46.	Fluconazole-Simvastatin	FLUCONAZOLE may result in increased simvastatin exposure.
47.	Fluticasone Propionate-Posaconazole	STRONG CYP3A4 INHIBITORS may result in increased fluticasone exposure.
48.	Fluticasone Propionate-Voriconazole	STRONG CYP3A4 INHIBITORS may result in increased fluticasone exposure.
49.	Ganciclovir-Tenofovir Alafenamide	GANCICLOVIR may result in increased tenofovir alafenamide exposure and an increased risk of tenofovir alafenamide-related adverse effects.
50.	Glipizide-Levofloxacin	Concurrent use of ANTIDIABETIC AGENTS and FLUOROQUINOLONES may result in changes in blood glucose and increased risk of hypoglycemia or hyperglycemia.
51.	Insulin Pork Regular-Levofloxacin	Concurrent use of ANTIDIABETIC AGENTS and FLUOROQUINOLONES may result in changes in blood glucose and increased risk of hypoglycemia or hyperglycemia.
52.	Isoniazid-Rifampin	Concurrent use of RIFAMPIN and ISONIAZID may result in hepatotoxicity.
53.	Itraconazole-Rifampin	STRONG CYP3A4 INDUCERS may result in reduced itraconazole exposure and reduced efficacy of itraconazole.
54.	Itraconazole-Ritonavir	Concurrent use of ITRACONAZOLE and RITONAVIR may result in increased ritonavir and/ or itraconazole exposure and an increased risk of itraconazole/ ritonavir-related adverse effects.
55.	Itraconazole-Salmeterol Xinafoate	STRONG CYP3A INHIBITORS may result in increased salmeterol exposure and an increased risk of salmeterol-related adverse effects (including cardiovascular adverse effects).
56.	Itraconazole-Simvastatin	ITRACONAZOLE may result in increased simvastatin exposure and an increased risk of myopathy and rhabdomyolysis.
57.	Itraconazole-Tamsulosin Hydrochloride	ITRACONAZOLE may result in increased tamsulosin exposure.
58.	Lansoprazole-Rilpivirine Hydrochloride	PROTON PUMP INHIBITORS may result in reduced rilpivirine exposure and an increased risk of diminished therapeutic effect of rilpivirine.
59.	Losartan Potassium-Sulfamethoxazole/Trimethoprim	Concurrent use of TRIMETHOPRIM and SELECTED POTASSIUM-SPARING DRUGS may result in increased risk of hyperkalemia.
60.	Nevirapine-Rifampin	RIFAMPIN may result in decreased nevirapine serum concentrations and possible loss of nevirapine efficacy.
61.	Nirmatrelvir/Ritonavir-Rifampin	RIFAMPIN may result in reduced nirmatrelvir/ ritonavir exposure and a potential for loss of virologic response and possible resistance.
62.	Omeprazole-Rilpivirine Hydrochloride	PROTON PUMP INHIBITORS may result in reduced rilpivirine exposure and an increased risk of diminished therapeutic effect of rilpivirine.
63.	Posaconazole-Simvastatin	POSACONAZOLE may result in increased simvastatin plasma concentrations and increased risk of myopathy or rhabdomyolysis.
64.	Posaconazole-Tamsulosin Hydrochloride	STRONG CYP3A4 INHIBITORS may result in increased tamsulosin exposure.
65.	Pyrazinamide-Rifampin	Concurrent use of PYRAZINAMIDE and RIFAMPIN may result in severe hepatic injury.
66.	Rifampin-Rilpivirine Hydrochloride	RIFAMPIN may result in reduced rilpivirine plasma concentrations and risk of diminished therapeutic effect of rilpivirine.
67.	Rifampin-Ritonavir	RIFAMPIN may result in reduced ritonavir exposure.
68.	Rifampin-Tenofovir Alafenamide	RIFAMPIN may result in decreased tenofovir alafenamide exposure, loss of therapeutic effect, and increased risk of resistance.
69.	Rifampin-Voriconazole	RIFAMPIN may result in reduced systemic exposure to voriconazole.
70.	Ritonavir-Rosuvastatin Calcium	Concurrent use of RITONAVIR and ROSUVASTATIN may result in an increased risk of myopathy, including rhabdomyolysis.
71.	Ritonavir-Salmeterol Xinafoate	STRONG CYP3A INHIBITORS may result in increased salmeterol exposure and an increased risk of salmeterol-related adverse effects (including cardiovascular adverse effects).
72.	Ritonavir-Simvastatin	Concurrent use of RITONAVIR and SIMVASTATIN may result in an increased risk of myopathy or rhabdomyolysis.
73.	Ritonavir-Tamsulosin Hydrochloride	STRONG CYP3A4 INHIBITORS may result in increased tamsulosin exposure.
74.	Ritonavir-Voriconazole	Concurrent use of RITONAVIR and VORICONAZOLE may result in decreased plasma concentrations of voriconazole with high-dose and, to a lesser extent, low-dose ritonavir, and risk of decreased voriconazole efficacy.
75.	Salmetreol Xinafoate-Voriconazole	STRONG CYP3A INHIBITORS may result in increased salmeterol exposure and an increased risk of salmeterol-related adverse effects (including cardiovascular adverse effects).
76.	Simvastatin-Voriconazole	VORICONAZOLE may result in increased plasma concentrations of simvastatin.
77.	Tamsulosin-Voriconazole	STRONG CYP3A4 INHIBITORS may result in increased tamsulosin exposure.

## Discussion

During the height of the COVID-19 pandemic, leading tertiary-care hospitals were at the forefront of the battle against this deadly infection. CNMI, under the administration of the Faculty of Medicine Ramathibodi Hospital, Mahidol University, played the leading role in caring for severe cases of COVID-19 infections in Thailand. Situated in the Samut Prakan province, east of Bangkok where large industrial factories are located, CNMI has become the major institute providing medical care for patients with COVID-19, with extensive experience in dealing with the original Wuhan strain, and the Alpha, Delta, and, most recently, Omicron variants [11]. More than half of severely ill patients, who commonly had multiple chronic conditions, went on to develop long COVID, which required long-term pharmacological interventions. Therefore, this study was performed to determine potential DDIs among frequently prescribed medications in long COVID. Because electronic databases are commonly used as the sources for DDI detection, we employed the two most commonly used databases, Micromedex and Drugs.com, in our analysis. It is common knowledge that different databases use different sources of primary information. As a result, discordance or disagreement among databases can be expected. These discrepancies can sometimes lead to potential miscommunication between healthcare professionals and their patients.

There were approximately 3,000 possible pairs of frequently prescribed medications in long COVID. The two electronic databases reported 488/3,000 (16%) pairs of potential DDIs. This value is between our two previous potential DDI reports of antiretrovirals/antimicrobials (3%–4%) and metabolic syndrome medications (18%–20%) [12, 13]. Considering the severity of potential DDIs reported by Micromedex, 191/271 pairs (70%) were reported as contraindicated or major in severity, compared with 70% for antiretrovirals/antimicrobials and 20% for metabolic syndrome medications. The percentage of potential DDIs among long COVID medications seems to be higher than potential DDIs of antiretroviral/antimicrobials (16% vs 3%–4%), and the degree of severity of potential DDIs among long COVID medications seems to be higher than metabolic syndrome medications (70% vs 20%). Therefore, healthcare providers should be aware of the high frequency and serious severity of potential DDIs among frequently prescribed medications for long COVID.

Metabolic syndrome significantly increases the severity of COVID-19 infection, and metabolic syndrome can progress after serious COVID infection [14]. The three primary presentations of metabolic syndrome are hypertension, dyslipidemia, and diabetes mellitus, each of which requires separate pharmacological interventions to treat [15]. Calcium channel blockers, angiotensin-converting enzyme (ACE) inhibitors, and angiotensin receptor blockers are commonly prescribed to manage hypertension. Statins are the main drugs for dyslipidemia management. A variety of both classical agents (i.e., sulphonylureas and metformin) and newer agents (i.e., incretin-based therapies and sodium-glucose cotransporter-2 inhibitors) are recommended by various international guidelines for the treatment of diabetes mellitus [16]. Calcium channel blockers such as amlodipine and most dihydropyridine calcium channel blockers are metabolized by hepatic CYP3A4, and thus are contraindicated for use with CYP3A4 inhibitors because they would increase the plasma amlodipine concentration [17, 18]. In addition, amlodipine can delay the metabolism of other agents requiring CYP3A4 activation such as simvastatin [18]. Hence, co-administration of amlodipine and simvastatin is contraindicated and might increase systemic exposure to simvastatin, resulting in the risk of myopathy or rhabdomyolysis [19]. On the contrary, clopidogrel is a popular antiplatelet drug that requires CYP hydroxylation to obtain active metabolites. Concomitant use of clopidogrel and CYP inhibitors, especially inhibitors of CYP2C19 such as omeprazole, might decrease the formation of active metabolites and reduce platelet inhibition [20]. Another major concern is medications used to treat benign prostatic hyperplasia (BPH) in elderly men. Most patients with BPH need medication to improve urinary flow, especially α_1A/D_ antagonists such as tamsulosin. Most α_1A/D_ antagonists require CYP inactivation by hydroxylation or dealkylation reactions. Co-administration of tamsulosin and other potent CYP3A4 inhibitors could increase plasma concentration of tamsulosin and lead to severe hypotension or priapism [21]. These examples of DDIs indicate that healthcare providers should carefully evaluate benefit/risk especially for potentially contraindicated/major DDIs when treating patients with long COVID.

Because long COVID commonly presents with respiratory symptoms, commonly prescribed agents include cough suppressants, bronchodilators, and corticosteroids. The respiratory complications in long COVID become significant in patients with pre-existing respiratory-related conditions, namely rhinitis, asthma, and tuberculosis. Certain agents are used to treat long COVID and respiratory symptoms such as asthma, while tuberculosis would require other agents to kill the bacterium. Based on the results of the study, potent corticosteroids such as inhaled budesonide or fluticasone are substrates of CYP3A4. Co-administration of these inhaled corticosteroids with CYP inhibitors might increase systemic exposure to potent corticosteroids, resulting in increased corticosteroid toxicity risks [22]. Dose reduction of inhaled corticosteroids or a switch to less potent corticosteroids could be useful strategies to prevent toxicity from potent inhaled corticosteroids.

Patients with COVID-19 in developing countries, where the prevalence of tuberculosis is high, represent a clinical challenge. Tuberculosis generally requires long-term use of multiple antimycobacterial drugs. A number of these agents are known to possess strong CYP inhibition and/or induction [23]. Rifampicin is the major culprit drug leading to DDIs that are considered contraindicated and major. Rifampicin is well recognized as a potent CYP inducer. Concomitant use of rifampicin with other medications metabolized by CYPs may lead to a drastic decrease in systemic exposure to those drugs. For example, the use of rifampicin with amlodipine, atazanavir, and voriconazole was contraindicated or major. A several fold decrease in the systemic exposure of these drugs has been documented in the literature [24–27]. Dose adjustment and monitoring of pharmacodynamics should be conducted or rifampicin should be switched to rifabutin may represent a safer alternative where possible.

Another challenging scenario includes the presence of chronic viral infection (such as human immunodeficiency virus [HIV]) or an existing fungal infection (such as COVID-associated pulmonary aspergillosis [CAPA]). Some antiretrovirals and antifungals have high potential for DDIs. This is based on the fact that most protease inhibitors and azole antifungals are mainly metabolized by CYPs and possess CYP inhibitory potential [28, 29]. Concomitant use of atazanavir, darunavir, ritonavir, and other drugs mainly metabolized by CYPs such as statins was classified as contraindicated or major by both electronic databases. The protease inhibitors could reduce metabolism of statins, leading to increased systemic exposure that results in rhabdomyolysis or myopathy. Monitoring creatine kinase levels, lowering the statin dosage, or switching to less potent HMG-CoA reductase inhibitors are possible management methods. Interestingly, there are four approved antivirals for COVID treatment in Thailand–favipiravir, remdesivir, molnupiravir, and nirmatrelvir/ritonavir. Recently, re-infections by different variants of SARS-CoV-2 have occurred frequently. As a result, these anti-SARS-CoV-2 medicines were included in this DDI analysis of frequently prescribed medications in long COVID except favipiravir. This drug was not recognized by both electronic databases. Favipiravir, one of the nucleoside analogues primarily designed to treat influenza in Japan, blocks RNA-dependent RNA polymerase (RdRP) of various viruses, including SAR-CoV-2, *in vitro* [30]. Remdesivir and molnupiravir are nucleoside analogues that have activity at RdRP of SARS-CoV-2 and minimal effects on CYP isoforms. The only concern is a combination of nirmatrelvir and ritonavir. Nirmatrelvir is active against main proteases, which is a viral 3C-like (3CL) protease that cleaves the 2 viral polyproteins and has a major role in SARS-CoV-2 replication [31]. Ritonavir is a protease inhibitor that is used as the booster for other protease inhibitors, including HIV protease inhibitors (atazanavir and darunavir) and nirmatrelvir. Nirmatrelvir/ritonavir are both commonly metabolized by CYPs, and ritonavir also shows moderate to strong CYP inhibitory activity. In addition, ritonavir can be combined with nirmatrelvir to boost systemic exposure of nirmatrelvir for better anti-SARS-CoV-2 activity. Both Micromedex and Drugs.com include a large number of contraindicated/major for concomitant uses of nirmatrelvir/ritonavir with other drugs that are highly dependent on CYP3A4 clearance. Additional information of real-world usage of the nirmatrelvir/ritonavir combination with other drugs requiring hepatic clearance through CYP metabolism is urgently required to help bring awareness of potential DDIs related to these agents.

The present study has several limitations. First, the frequently prescribed medications investigated do not cover all agents that are potentially used to treat long COVID. Second, reports of potential DDIs are constantly evolving. As a result, new potential DDIs, changes in severity classification, and changes in underlying mechanisms by both electronic databases are expected in the future. Third, the lack of reported DDIs does not necessarily mean there is an absence of risks for DDIs, and several DDI mechanisms are still unknown or have not been determined. In addition, potential DDIs in long COVID can vary individually depending on the patient’s conditions and underlying diseases. As a result, patient consultation and direct communication between patients and healthcare professionals remain paramount to maximize benefits and minimize risks in managing patients with long COVID.

## Conclusion

There are a large number of potential DDIs among frequently prescribed medications for patients with long COVID according to Micromedex and Drugs.com. There was slight agreement of the severity classification between the two electronic databases as determined by Fleiss’ kappa. The main mechanistics of potential DDIs are CYP inhibition and induction. Clinicians should be familiar with these DDIs, especially the serious DDIs that can result in harm to the patients. Antiretrovirals, antifungals, anti-tuberculosis drugs, statins, and corticosteroids are among the top five culprits for DDIs. Careful evaluation of DDIs when managing patients with long COVID, especially those with multiple chronic conditions requiring polypharmacy, should be implemented to promote safe and effective treatment plans for the patients.

## Abbreviations

AIDS: Acquired Immunodeficiency Syndromes
AUC: Area Under Curve
BPH: Benign Prostatic Hyperplasia
CNMI: Chakri Naruebodindra Medical Institute
COVID-19: Coronavirus Disease 2019
CYPs: Cytochrome P450s
DDIs: Drug-Drug Interactions
HIV: Human Immunodeficiency Virus
NLEM: National List of Essential Medicines
NNRTIs: Non-Nucleoside Reverse Transcriptase Inhibitors
SARS-CoV-2: Severe Acute Respiratory Syndrome Coronavirus 2
